# Ribotin: automated assembly and phasing of rDNA morphs

**DOI:** 10.1093/bioinformatics/btae124

**Published:** 2024-03-05

**Authors:** Mikko Rautiainen

**Affiliations:** Institute for Molecular Medicine Finland (FIMM), Helsinki Institute of Life Science (HiLIFE), University of Helsinki, Helsinki, Finland

## Abstract

**Motivation:**

The ribosomal DNA (rDNA) arrays are highly repetitive and homogenous regions which exist in all life. Due to their repetitiveness, current assembly methods do not fully assemble the rDNA arrays in humans and many other eukaryotes, and so variation within the rDNA arrays cannot be effectively studied.

**Results:**

Here, we present the tool ribotin to assemble full length rDNA copies, or *morphs*. Ribotin uses a combination of highly accurate long reads and extremely long nanopore reads to resolve the variation between rDNA morphs. We show that ribotin successfully recovers the most abundant morphs in human and nonhuman genomes. We also find that genome wide consensus sequences of the rDNA arrays frequently produce a mosaic sequence that does not exist in the genome.

**Availability and implementation:**

Ribotin is available on https://github.com/maickrau/ribotin and as a package on bioconda.

## 1 Introduction

The ribosomal DNA (rDNA) arrays are highly repetitive genomic regions coding for the 5.8s, 18s and 28s rRNAs of the ribosomes, the molecular machinery used in translating proteins. Ribosomes are ubiquitous to all life and are necessary for cellular function, and might be a remnant of a hypothetical ‘RNA world’ before life evolved DNA (Cech 2012). Heterogeneity within ribosomes has been hypothesized to give rise to specialized ribosomes with differential effects on protein synthesis (Xue and Barna 2012). A recent review (Hall *et al.* 2022) listed associations between variation in rDNA and cancer, schizophrenia, intellectual disability and aging in humans, but noted that the associations are uncertain due to small sample sizes and limited methods.

Some manual efforts to assemble rDNA arrays in humans and other organisms have been done (Hori *et al.* 2021, Ding *et al.* 2022, Nurk *et al.* 2022), but to the best of our knowledge there is no automated tool to perform rDNA assembly. Many aspects of the rDNA arrays, from basic structural issues such as the prevalence of inverted sequences and nontandem repeats to more medically relevant issues such as the functional impact of rDNA variation, remain open questions due to the lack of methods to assemble rDNA arrays (Hall *et al.* 2022). An automated method to assemble rDNA arrays would enable studies to look at large numbers of human genomes and investigate these questions.

Due to their repetitiveness and homogeneity, the rDNA arrays are the only remaining regions in human genomes which are inaccessible with existing genome assembly methods. Although current state of the art genome assembly tools (Cheng *et al.* 2023, Rautiainen *et al.* 2023) can assemble nearly everything in human genomes, they do not assemble the rDNA arrays completely outside of a few cases when the rDNA arrays are particularly short.

In humans, the rDNA arrays are located in chromosomes 13, 14, 15, 21, and 22 and are composed of a few hundred copies of an approximately 45 kbp repeat unit arranged in tandem repeats. These repeat units are highly similar, with each array typically having dozens of identical or near-identical repeat units. Copies within the same chromosome are more similar than copies in different chromosomes, and there is chromosome specific variation which enables different rDNA copies to be assigned to different chromosomes (Nurk *et al.* 2022).

The release of the CHM13 telomere-to-telomere genome in 2022 (Nurk *et al.* 2022) provided the first chromosome resolved assembly of the rDNA arrays of one human genome. The rDNA arrays were manually assembled, and due to the difficulty of assembling rDNA arrays, the arrays were filled with model sequence corresponding to the most common repeat units per chromosome duplicated according to their estimated copy counts in arbitrary order. The assembly resolved chromosome specific *morphs*. A morph is the sequence of one complete repeat unit which appears in the rDNA arrays once or multiple times. Although the CHM13 assembly (Nurk *et al.* 2022) is currently the most comprehensive assembly of rDNA arrays, it was the result of manual effort by many experts around the globe with the author contribution listing 17 individuals working on assembly, making such a project impractical to repeat for multiple genomes.

Here, we present the tool ribotin to automatically assemble rDNA morphs using a combination of long reads. Ribotin requires high accuracy long reads such as PacBio HiFi, and additionally very long reads such as ultralong nanopore reads (ONT) to resolve complete morphs. Since assemblers using HiFi reads sometimes separate the rDNA arrays in different chromosomes due to chromosome specific variation, this information can be used to perform rDNA assembly in a chromosome specific manner. Ribotin has integration with the assembly tool verkko (Rautiainen *et al.* 2023) to assemble rDNA morphs per chromosome. Ribotin also has a mode to run without a verkko assembly using only a related reference rDNA sequence.

We test ribotin on human, gorilla, *Caenorhabditis elegans* and *Arabidopsis thaliana* genomes, and find that it successfully resolves the morphs of the CHM13 cell line matching previous assembly, resolves rDNA morphs in nonhuman genomes, and in the case of *C.elegans* and *A.thaliana* discovers nearly all variation in the rDNA arrays. We also perform a small experiment on the HG002 genome showing how the morphs can be used for downstream analysis.

## 2 Materials and methods

Ribotin requires highly accurate long reads, such as PacBio HiFi or Oxford Nanopore Duplex reads, in order to build a genome wide consensus rDNA sequence and detect variation in the rDNA. In addition, reads long enough to span complete rDNA units are required for resolving complete rDNA morphs.

Ribotin has two modes: a *reference-based* mode (*ribotin-ref*), and *verkko-based* mode (*ribotin-verkko*). In ribotin-ref, a reference rDNA sequence is used to recruit HiFi reads. In ribotin-verkko, a reference rDNA sequence is used to detect unresolved rDNA clusters in a verkko (Rautiainen *et al.* 2023) assembly, and the hifi reads assigned to those clusters are used. The benefit given by this depends on how well the clusters are separated in the verkko assembly, with the best results when all rDNA arrays are in separate tangles and no benefit when all rDNA arrays are in the same tangle. In addition, in ribotin-verkko the user may manually select rDNA nodes instead of using a reference sequence to detect them. Ribotin-verkko is intended to be used in cases where verkko fails to resolve the rDNA arrays, which is usually the case in human genomes. Ribotin-ref handles all rDNA hifi reads in one go, while ribotin-verkko processes each distinct rDNA tangle separately. The reference sequence does not need to be fully assembled or contain any full length morphs as long as it contains most of the rDNA k-mers present in the genome.

**Figure 1.**
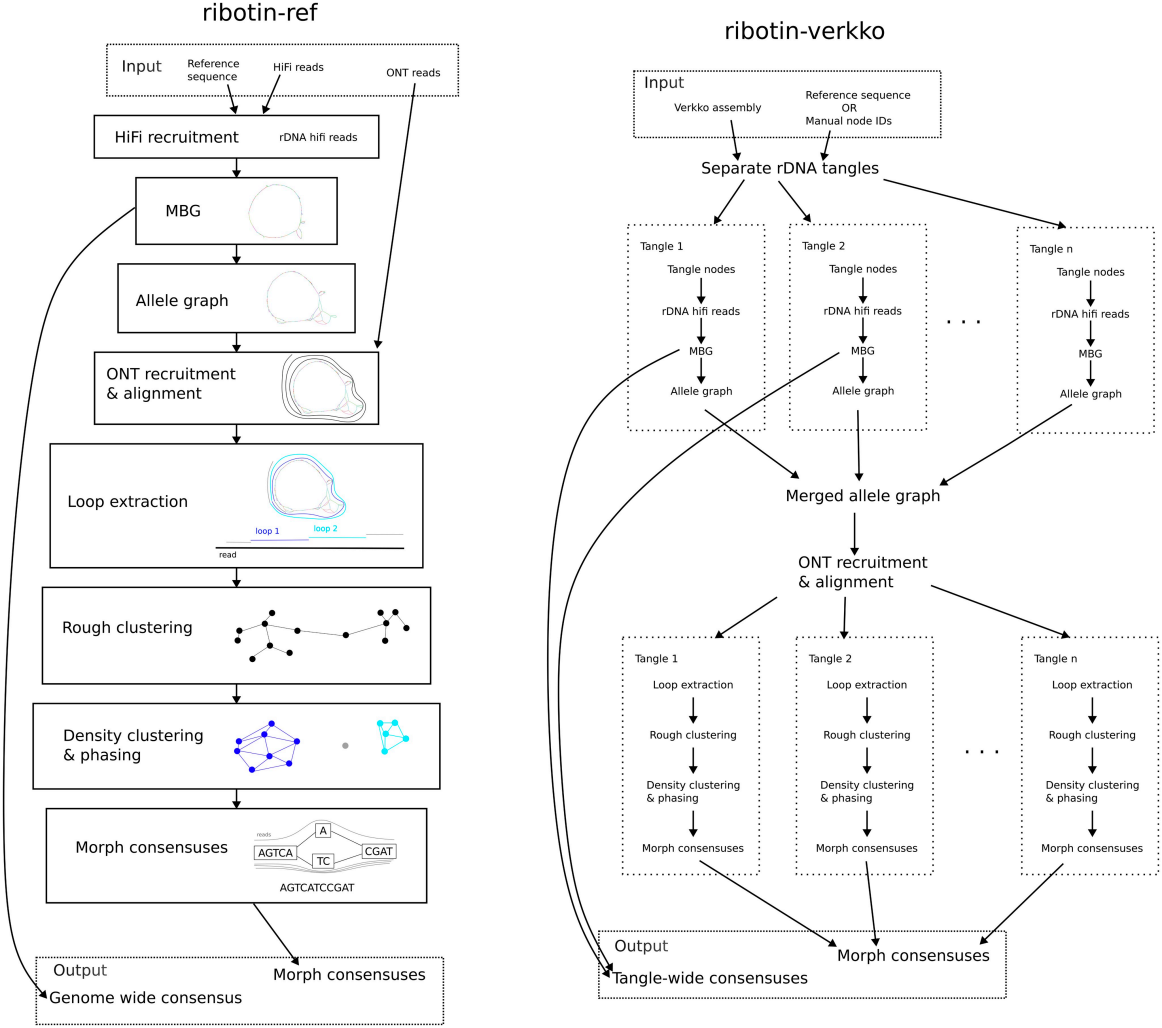
Overview of ribotin. Left: the reference based *ribotin-ref* mode. A reference sequence is used to recruit HiFi reads, which are then assembled with MBG and processed into the *allele graph* which represents all variation present within the rDNA. Then, ONT reads are recruited based on the k-mers of the allele graph and aligned to the allele graph. Loops are extracted from the alignments, which are then clustered to morphs. Finally, a consensus sequence is found for each cluster. Right: the verkko based *ribotin-verkko* mode. A verkko assembly is required, along with either a reference sequence used for detecting rDNA tangles or a manual assignment of node IDs to rDNA tangles. The HiFi reads are assigned to rDNA tangles based on their locations in the verkko assembly. Then, a pipeline similar to ribotin-ref is used per tangle. The ONT reads are recruited and aligned simultaneously to all allele graphs but otherwise the steps are the same as ribotin-ref.

Figure 1 shows an outline of ribotin and Supplementary Note SA has detailed information about the individual steps. Ribotin first uses MBG (Rautiainen *et al.* 2020b) to build a graph which represents all variation within the rDNA using the highly accurate long reads. Then ultralong ONT reads are aligned to the graph with GraphAligner (Rautiainen *et al.* 2020a) and the alignment paths are used to extract *loops*, complete sequences of a single repeat unit, from the alignment paths. The loops are clustered based on their pairwise edit distances, first to *rough clusters* using a union-find data structure and next into *density clusters* using the DBscan (Ester *et al.* 1996) algorithm. DBscan requires a parameter ϵ which determines how similar loops are clustered. ϵ is essentially a similarity threshold for merging clusters, with higher ϵ leading to less similar loops being assigned to the same cluster and therefore leading to a lower morph resolution, while lower ϵ splits the clusters more aggressively and leads to better morph resolution. Ribotin estimates the ϵ parameter from pairwise loop edit distances, which gives a rough approximation of how well the morphs were separated, with lower ϵ indicating better separation. The clustered loops are then used to build a consensus for each cluster, and the clusters are output as the morphs.

## 3 Results

We ran ribotin-ref and ribotin-verkko on several genomes using simulated and real data. Comparing sets of morphs requires finding similar morphs. When we compare morphs, we align them to each others with minimap2 (Li 2018) and say that two morphs *match* if they have an alignment with at least 99% identity covering at least 99% of both morphs. We used the parameters ‘-c -x asm5’ for minimap2.

We used ribotin version 1.2, verkko (Rautiainen *et al.* 2023) version 1.4.1, MBG (Rautiainen *et al.* 2020b) version 1.0.16, GraphAligner (Rautiainen *et al.* 2020a) version 1.0.18 and minimap2 (Li 2018) version 2.26-r1175. Source code for running the experiments is available at https://github.com/maickrau/ribotin_paper_experiments.

### 3.1 Human rDNA simulation

We evaluated the accuracy of ribotin on simulated data. We generated simulated rDNA arrays by generating mosaics of the nine so far assembled complete human rDNA morphs and then inserting random mutations. Supplementary Note SB.1 describes the details of generating the simulated rDNA arrays. The morphs have an average divergence of 4.4%, of which 4.2% is due to the mosaic sampling of existing morphs and 0.2% is additional random mutations. We then simulated HiFi and ultralong nanopore reads from the simulated array with pbsim3 (Ono *et al.* 2022). Then, we ran ribotin-ref on the simulated reads and compared the results to the simulated ground truth.

We evaluated three metrics: first, the sensitivity and specificity of ribotin-ref morphs against the ground truth, second, correlation between the ribotin-ref estimated coverages and the ground truth copy counts, and third, the average error rate of the ribotin-ref morphs. Supplementary Note SB.1 describes the details of how these metrics were measured. We repeated the simulation 20 times, generating a new simulated rDNA array every time.

The Pearson correlation between ribotin-ref assigned ONT coverage and simulated ground truth copy count was 0.94. The coverage of ribotin-ref morphs was on average 4.9 times the copy count. For comparison, the expected coverage at the simulated ONT read length distribution is 7.8 times copy count assuming no sequencing bias. Overall sensitivity was 87% and specificity was 87%. For morphs with a ground truth copy count at least 5 or ribotin coverage at least 30, sensitivity was 93% and specificity was 94%. On average the morphs had 162 mismatches corresponding to 0.36% error rate, composed of 23% homopolymer indels, 52% microsatellite indels and 25% for all other errors.

The results of the simulation show that ribotin can reliably recover high copy count morphs. Although this simulation is based on all known complete rDNA morphs and it includes the variation present in them, it has limitations. The mutations inserted after sampling the mosaic sequences were random, which might not be biologically realistic. In addition the read simulation might not accurately generate the systematic errors and biases present in real reads. In particular, real HiFi reads have systematic coverage biases in rDNA regions which we did not observe in the pbsim3 simulated reads. The simulation also represents only the rDNA variation currently known, which is likely only a small fraction of the full range of variation present in human rDNA, while using all such variation for simulating each rDNA array. This means the simulated rDNA arrays likely have less variation than the population of human rDNA arrays, while simultaneously a single simulated rDNA array has more variation than a single real rDNA array.

### 3.2 CHM13

The CHM13 assembly (Nurk *et al.* 2022) is so far the only human assembly with resolved rDNA sequences. We used the existing assembly as the ground truth to evaluate ribotin’s accuracy. We used the same HiFi and ONT data that was used to generate the assembly, consisting of approximately 35× haploid coverage HiFi and 120× ONT.

First we checked if current hybrid long read assemblers successfully recover the CHM13 major morphs. We ran verkko (Rautiainen *et al.* 2023) and hifiasm (Cheng *et al.* 2023) on the same HiFi and ONT reads. Both assemblers failed to assemble the rDNA arrays fully as expected, and had unresolved tangles at the locations of the rDNA arrays. Then we aligned the major morphs to the assembled contigs with minimap2 (Li 2018) and filtered to alignments which cover at least 99% of the major morph with at least 99% identity. Verkko did not have a contig which fully contained the morphs chr15b or chr21b, and the morph chr13 was represented in seven different locations. Hifiasm represented the morphs in multiple locations in a way that is inconsistent with the ground truth copy counts, e.g. the chr14 morph (ground truth copy count 12) was located in 7 places while the chr21a morph (ground truth copy count 15) was located in one place. Getting accurate copy counts of the morphs from the assembled contigs is not trivial due to the contigs being represented in multiple places including different contigs, contigs sometimes having copy counts >1, and morphs straddling between different contigs such that the start and end of a morph are in different contigs.

In ribotin-verkko, all the chromosomes were assigned to the same tangle due to the verkko assembly containing short spurious nodes connecting the rDNA tangles. Even though visual inspection with bandage shows that the five rDNA arrays are separate (Supplementary Fig. S1), there are short spurious nodes connecting them which causes the tangle detection to consider all five arrays one large tangle. We additionally ran ribotin-verkko in a chromosome specific manner by manually picking the nodes of the five arrays. We refer to the results with the manually picked nodes as the *chromosome-specific ribotin-verkko* results.

In ribotin-ref, the ONT reads successfully phased out chromosome specific morphs. Supplementary Figure S5 shows how the ribotin-ref morphs match the automatic ribotin-verkko morphs. Since all five rDNA arrays were assigned to a single tangle, the results are basically the same between ribotin-ref and ribotin-verkko, with one ribotin-ref morph split into two ribotin-verkko morphs since the HiFi read recruitment is not completely identical. Supplementary Figure S6 shows how the ribotin-ref morphs match the chromosome-specific ribotin-verkko morphs. We observed that the chromosome-specific ribotin-verkko resulted in more morphs than ribotin-ref (61 versus 36) due to successfully separating out high similarity low copy count morphs. The ϵ parameter chosen by ribotin was highly dependent on how many chromosomes a tangle contained: ribotin-ref which contained all five chromosomes in the same tangle had ϵ=80 while the chromosome specific ribotin-verkko had ϵ=5 in all five tangles. The results in Supplementary Fig. S6 support this, showing that the ribotin-ref morphs sometimes match several ribotin-verkko morphs, with the total coverage approximately matching. In a few cases the morph matchings formed a clique due to resolving morphs which are >99% similar and therefore align to each others at 99% similarity. However, there were morphs even within the same chromosome which were <99% similar to each others. The ribotin-ref morphs ranged in size from 38 to 49 kbp, with the average length weighted by coverage being 45 064 bp.

We compared the morphs generated by ribotin-ref to the rDNA model sequences resolved by the CHM13 assembly (Nurk *et al.* 2022). Since the CHM13 assembly rDNA model sequence only includes major morphs, even though the assembly process produced some low copy count morphs [(Nurk *et al.* 2022) Fig. S12 panel c], we limit the comparison to the major rDNA morphs present in the assembly. We extracted the major rDNA morphs from the CHM13 assembly, and assigned a copy count based on how many times the exact same morph appears in the assembly. We refer to the previously resolved morphs as CHM13 morphs.

**Figure 2.**
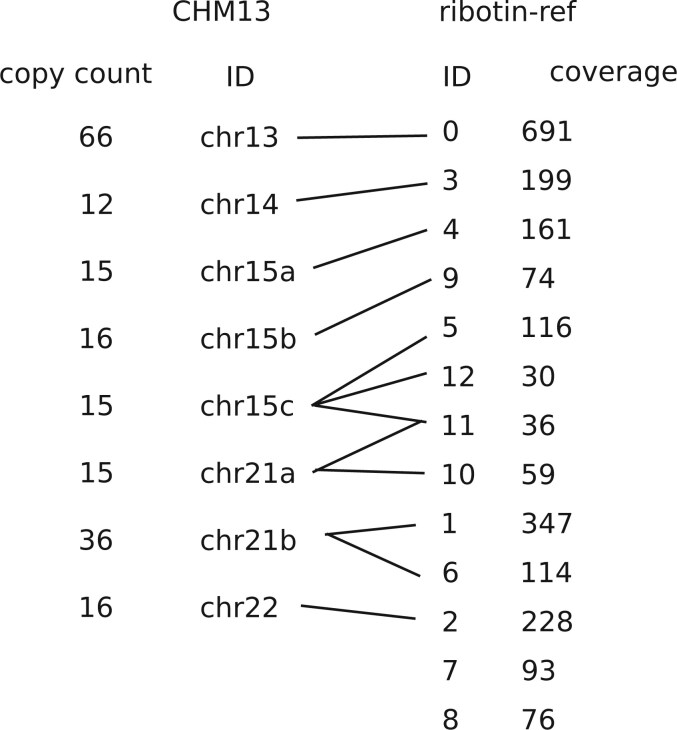
Comparison of the CHM13 major morphs and ribotin-ref morphs. All matching morphs are connected by a line. CHM13 morphs are ordered by ID and ribotin-ref morphs are ordered for clarity. Morphs with coverage <30 are not shown.

Figure 2 shows how CHM13 morphs and ribotin-ref morphs match each others. All CHM13 morphs are recovered by ribotin. Some of the CHM13 morphs (chr15c, chr21a, chr21b) match multiple ribotin morphs due to ribotin separating similar but not identical morphs. The highest coverage ribotin morph not found in the CHM13 major morphs was id 7 with coverage 93. Since the CHM13 morphs only included the most abundant morphs, it is expected that some low copy count ribotin morphs are not found in the CHM13 morphs. This shows that the morphs recovered by ribotin match the previous assembly. However, we observed a relatively high average error rate, with matching morphs typically having 100–200 mismatches (0.2%–0.4% error rate). The error rate was computed from the number of edits of the minimap2 alignment of the highest coverage ribotin morph which matches the CHM13 morph.

We also compared the morphs generated by the manual chromosome specific mode to the CHM13 morphs. All CHM13 morphs were recovered. In addition, the error rates were low. The most abundant morph in chromosomes 13, 14, 21, and 22 ranged between 0.009% and 0.04% error rate, but the second most abundant morph of chr21 as well as the morphs in the more variable chr15 (chr15a, chr15b, chr15c, chr21b) had higher error rates from 0.11% to 0.46%. This might be due to MBG collapsing microsatellite variation within each chromosome.

The main difference between the manual chromosome specific mode and the automatic ribotin-ref mode is that the manual mode has a noticably higher consensus accuracy. The manual mode also separated out more low copy count morphs which are highly similar to the abundant morphs, but the high copy count morphs were recovered in both modes. This shows that while separating out the rDNA arrays by chromosome is helpful for getting best results, it is not necessary for recovering the most abundant morphs.

We observed that the genome wide consensus in ribotin-ref only matched to four morph with 99% identity, and did not match any of the higher coverage ribotin morphs or the CHM13 morphs. The highest identity match among those four morphs had only 99.5% identity. This is due to the presence of chromosome specific variants: all of the chromosomes have some variation present in nearly every copy in the same chromosome but missing from other chromosomes (Nurk *et al.* 2022), but the single genome wide consensus lacks any of these variants and therefore does not actually correspond to any sequence present in the genome. On the other hand, the chromosome wide consensuses generated by the manual chromosome specific mode do match the highest coverage morphs in their chromosomes with very low error rates (0, 4, 8, 0, and 0 mismatches corresponding to error rates of 0%, 0.009%, 0.02%, 0%, and 0% for chr13, 14, 15, 21, and 22, respectively) and therefore correspond to genomic sequence. All of the errors in the chromosome wide consensuses were either homopolymer or microsatellite indels.

### 3.3 Human rDNA assembly

We ran ribotin on the human sample HG002. Since this sample does not have a previous rDNA assembly, there is no ground truth to compare to. We compare the consistency of the ribotin-ref morphs to the ribotin-verkko morphs. The sample had 30× HiFi coverage and 60× ONT coverage.

On the HG002 assembly, the CHM13 based reference successfully recruited the relevant HiFi reads and the nodes in the verkko assembly. Ribotin-verkko detected two tangles in the verkko assembly graph. Again a visual inspection with bandage (Supplementary Fig. S2) suggests that the tangles should be more separated, but short nodes connecting the tangles cause them to be merged to the same tangle. In this case the bandage plot is too ambiguous to clearly separate the ten rDNA arrays, so we did not attempt to manually select the rDNA nodes for a chromosome specific assembly.

Ribotin-verkko had two tangles with ϵ 95 and 5, while ribotin-ref used ϵ=134. The morphs are roughly consistent between the two modes. One ribotin-verkko morph was split into two in ribotin-ref, and some ribotin-ref morphs were split in ribotin-verkko. This shows that tangles with fewer chromosomes are better resolved, although the difference is small in this case due to only one array being separated in ribotin-verkko. This shows that the CHM13 reference is similar enough to be used for recruiting rDNA reads in human genomes. The ribotin-ref morphs ranged in size from 41 to 49 kbp, with the average length weighted by coverage being 44 901 bp. Supplementary Figure S3 shows how the morphs match between ribotin-verkko and ribotin-ref.

We also ran ribotin-ref using a lower coverage dataset with one cell of HiFi from PacBio Sequel II and one cell of ultralong ONT from Promethion, containing 10× HiFi and 35× ONT. We then compared the results to the full coverage ribotin-ref results. Curiously, the lower coverage dataset had an estimated ϵ=72, almost half of the full coverage dataset. Despite this, the higher coverage dataset still resolved the morphs slightly better. The two sets are roughly similar, showing that ribotin can recover abundant morphs even from one cell of HiFi and ONT each. Supplementary Figure S4 shows the results.

We again observed that the genome wide consensus is a poor match to the morphs. In ribotin-ref the most similar morph had merely 98.5% identity to the consensus. The smaller verkko tangle produced a consensus which does match its highest coverage morph with zero mismatches, and the consensus of the other verkko tangle matched a low coverage morph at approximately 99.5% identity.

### 3.4 Gorilla

We tested ribotin on a gorilla genome to test the performance on a nonhuman genome. In contrast to the human HG002, we observed that the default CHM13 based reference is not sufficient for recruiting rDNA hifi reads due to dissimilarities between human and gorilla rDNA sequences.

The results using the CHM13 reference misses much variation and most morphs. ϵ was estimated at 198, and all loops were clustered into a single morph. To solve this, we used MBG to build an assembly from the whole genome hifi reads, manually picked out the rDNA cluster contigs, and used those as the reference. This resulted in estimated ϵ=95 and produced 6 morphs with coverage ≥30. This shows that even in closely related species, there are differences between the rDNA sequences which need to be taken into account.

We also ran ribotin-verkko with the same MBG based reference. Verkko collapsed the rDNAs into a single tangle, resulting in no improvement over the reference based mode. The ribotin-ref morphs mostly ranged in size from 37 to 38 kbp with two exceptions, one morph with length 25 781 and coverage 6 and another with length 33 001 and coverage 10, with the average length weighted by coverage being 38 123 bp.

The genome wide consensus from ribotin-ref has an alignment identity of at most 99.5% to any morph.

The gorilla morphs were approximately 38 kbp long compared to the human 45 kbp. Ribotin was given 45 000 as the approximate morph size parameter, showing that slight differences between the estimated and real sizes do not matter.

### 3.5 Caenorhabditis elegans

*Caenorhabditis elegans* has a relatively short rDNA array with a morph length of approximately 7 kbp and copy counts estimated from dozens to hundreds. We ran ribotin-ref on two *C.elegans* strains (ALT1, ALT2) using HiFi data from Lee *et al.* (2023). The default CHM13 reference did not recruit any rDNA reads and we generated a species-specific reference with MBG. Since the hifi reads are longer than rDNA morphs (read N50 14 936 versus ∼7 kbp), they can contain entire rDNA morphs, which enables using high accuracy reads to obtain accurate morphs. We tested this by running ribotin-ref using the HiFi reads as both the hifi reads and ultralong ONT reads, and setting the maximum rough cluster difference to 10 edits and minimum ϵ as 1. The whole genome hifi dataset of ALT1 (respectively ALT2) has haploid coverage 15× (13×), with expected 8× (7×) coverage of complete single morphs, and expected 2.7× (1.5×) coverage of complete adjacent morphs. Ribotin estimated an average within-morph edit distance of 0 which resulted in ϵ=1. This produced two morphs for ALT1 with coverages 934 and 13, and one morph for ALT2 with coverage 2118. The main morphs of the two strains had identical sequences of length 7195 bp. The low coverage morph in ALT1 has length 3717 bp, with a 3478 bp deletion in the middle but otherwise exactly matching the high coverage morph.

Ribotin reported 23 SNPs for ALT2. We believe that at least five of them are genuine SNPs but we could not confirm if the rest are genuine variation, and suspect that at least some of them might be recurring sequencing errors. No other variants were found in ALT2. Supplementary Note SB.2 has details about the SNP variants.

The rDNA morph coverage was twice as high for ALT2 than ALT1, implying that these closely related strains had widely different copy counts. We checked whether this could be an artefact of ribotin by using two different methods to estimate the copy counts from the reads and found out that the different methods agree with ribotin, showing that the copy counts genuinely do vary by a factor of 2 between the strains. Supplementary Note SB.2 has details about the copy count estimation.

We believe that the combination of the morphs and reported SNPs contains nearly all rDNA variation present in these two strains, but some of the reported SNPs might be false positives. The morphs themselves do not represent all SNP variation, and some morphs which differ by only one or two SNPs were collapsed into the highest coverage morph. Homopolymer and microsatellite variation might remain unreported since ribotin’s first step collapses variation in homopolymers and microsatellites if there is no other variation nearby. Since there is no significant variation between the morphs, these results could be used to build a model sequence of the *C.elegans* rDNA similar to the CHM13 assembly (Nurk *et al.* 2022) by duplicating the morph an appropriate number of times, although this would require an accurate copy count estimate. For ALT2 this would be essentially a complete assembly of its rDNA, but for ALT1 it would leave the exact location of the 3478 bp deletion(s) unresolved.

### 3.6 Arabidopsis thaliana

We also ran ribotin on *A.thaliana* using hifi and ONT data from Wang *et al.* (2022). The *A.thaliana* rDNA is much shorter than human rDNAs at only approximately 10 kbp per morph. We again observed that the default CHM13 rDNA reference did not recruit any reads. We generated a whole genome hifi assembly using MBG, manually located the rDNA tangles by using blast (Altschul *et al.* 1990) to align the node sequences against the *A.thaliana* reference, and picked the node sequences in the tangle as the rDNA reference. We used the same node sequences in ribotin-verkko to detect the rDNA tangle. Ribotin-verkko detected only one tangle, resulting in essentially the same pipeline and same results as ribotin-ref.

Since the hifi reads are longer than rDNA morphs (read N50 14936), they can contain entire rDNA morphs, which enables using high accuracy reads to obtain accurate morphs. The whole genome hifi dataset has haploid coverage 170×, with expected 28× coverage of complete single morphs, and expected 0.35× coverage of complete adjacent morphs. We tested this by running ribotin-ref using the HiFi reads as both the hifi reads and ultralong ONT reads, and setting the maximum rough cluster difference to 10 edits and minimum ϵ as 1. Ribotin estimated an average within-morph edit distance of 0 which resulted in ϵ=1. This produced 54 distinct morphs with estimated copy count at least one (coverage at least 14), and two further morphs with coverage less than half the expected one copy coverage, for a total of 56 morphs. Using expected coverage of 28× and assuming no coverage bias, the morphs have a total estimated copy count of 765. HiFi sequencing has systematically lower coverage in the rDNA arrays (Nurk *et al.* 2022) and thus the real copy count is likely higher. The most abundant morph had a coverage of 5172, containing slightly over a quarter of the loops. The genome wide consensus sequence exactly matched the most abundant morph with no mismatches, and three other low coverage morphs matched the genome wide consensus with <5 mismatches, totaling about 5255 loops or 25% of the loops. This shows that despite the homogeneity of the rDNA arrays, this genome did not have a single morph which would account for the majority of the rDNA sequence. The morphs ranged in size from 9 to 12 kbp, with the average length weighted by coverage being 10 381 bp.

We believe these morphs contain nearly all rDNA variation that occurs in this *A.thaliana* individual as well as their relative abundances. Due to ribotin’s choice of ϵ=1, morphs which differ by one edit are collapsed together. We cannot rule out the possibility that low coverage morphs which differ by a single small variant are collapsed into other morphs, but morphs with two or more variants, or a single variant larger than 5 bp, are very likely separated. Although the HiFi reads are long enough to span individual rDNA morphs, they rarely span two or more morphs, and so this approach did not enable finding the exact order of the morphs. Even longer high accuracy reads such as ONT duplex might enable a similar approach to resolve the order of the morphs as well.

### 3.7 HG002 morph pangenome

**Figure 3.**
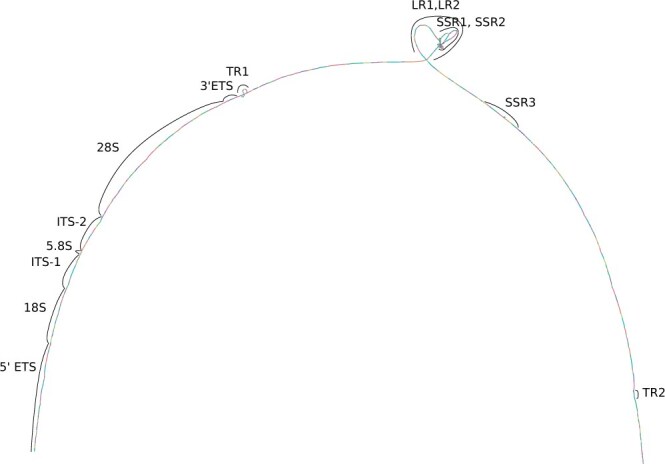
Bandage (Wick *et al*. 2015)  plot of the HG002 morph pangenome with some regions labeled. Supplementary Figure S9 has a larger version.

To show how the morphs might be used for studying variation within the rDNA arrays, we built a *morph pangenome* out of the HG002 ribotin-ref morphs. We used minimap2 (Li 2018) to align all the morphs against each others, then ran seqwish (Garrison and Guarracino 2023) to build a pangenome graph out of them. This produced a graph with 2671 nodes and 4009 edges. Figure 3 shows a bandage plot of the graph.

We observed that there was a considerable amount of variation even within just this one genome. The graph had 731 bubbles, each corresponding to one variant site, of which 371 had >2 alleles. The longest stretch without any variants was only 421 base pairs long. Supplementary Figure S8 shows some examples of variant sites.

We used GraphAligner to align the genome wide HG002 consensus sequence to the graph, and the sequences of the 18s, 5.8s, and 28s genes from the KY962518.1 rDNA reference sequence (Kim *et al.* 2018). The HG002 genome wide consensus sequence (which was not used in building the graph) aligned to the graph with zero mismatches as expected, taking a path which is a mosaic of different morphs without exactly matching any of them fully. Since the KY962518.1 rDNA reference sequence is from a different individual, the 18s, 5.8s, and 28s genes aligned to the graph with 0, 0, and 8 mismatches, showing that the reference contains some 28s variation not present in HG002. Using minimap2 to align the 18s, 5.8s, and 28s genes to the morphs instead of the graph, the highest identity alignments had 0, 0, and 21 mismatches.

Supplementary Table S1 counts the number of variants within the 18s, 5.8s, and 28s genes as well as the ITS-1, ITS-2, 3’ETS, and 5’ETS regions adjacent to the genes. There were also regions showing structural variation between the morphs (Fig. 3 TR1, LR1, LR2, SSR1, SSR2, SSR3) caused by different numbers of copies of the tandem repeat regions. Supplementary Figure S7 shows histograms of the lengths of the different loci, with different copy counts clearly visible in the repeats TR1, TR2, LR1, LR2, SSR1, SSR2, and SSR3. Although this is basically a toy experiment, it demonstrates how the morphs could be applied for downstream analysis.

### 3.8 Runtime

Table 1 shows the runtime of ribotin with each dataset and mode. Ribotin was given 8 threads in all runs. The runtime and peak memory was measured by the slurm scheduler’s ‘seff’ command. We see that the runtimes are modest and memory use is low, enabling ribotin to be easily ran on a large number of samples. Human samples can be processed in a few hours with just several Gb of memory. Curiously, the shorter *A.thaliana* genome required much more memory than human. This might be due to the very high coverage of the *A.thaliana* dataset. Note that the runtime only includes running ribotin and does not include the computational cost of running verkko, which is larger than ribotin by multiple orders of magnitude.

**Table 1.** Runtime of ribotin measured by *seff*.

Sample	Method	CPU-time	Wall time	RAM
		(h:m:s)	(h:m:s)	(Gb)
CHM13	Ribotin-ref	07:55:27	03:38:06	1.42
	Ribotin-verkko	07:43:31	03:53:33	2.23
	Ribotin-verkko (manual)	07:40:55	03:34:35	2.13
HG002	Ribotin-ref	05:02:06	02:56:09	2.65
	Ribotin-verkko	02:23:39	01:21:28	2.99
	Ribotin-ref (low coverage)	01:36:48	00:49:43	0.65
Gorilla	Ribotin-ref	05:12:35	01:52:28	0.58
	Ribotin-verkko	03:10:23	01:09:40	2.31
*A. thaliana*	Ribotin-ref	49:27:31	39:38:35	40.65
	Ribotin-verkko	49:27:08	42:02:35	8.85
	Ribotin-ref (HiFi)	06:35:27	03:25:23	40.58
*C. elegans*	ALT1 ribotin-ref	00:03:35	00:02:32	0.02
	ALT2 ribotin-ref	00:05:30	00:02:58	0.28

## 4 Discussion

Ribotin enables easy, automated rDNA morph assembly for humans. This can be used to generate model rDNA arrays for telomere-to-telomere assembly efforts, since large rDNA arrays are not assembled by current genome assemblers. Our recommendation for telomere-to-telomere assembly efforts is to use ribotin-verkko with manually chosen rDNA arrays in order to generate the most accurate and complete results. The downside of this is that it involves manual curation. For projects where manual curation is impractical, we suggest using either the automatic mode of ribotin-verkko or ribotin-ref, depending on if the pipeline already involves running verkko. If the project is about nonhumans, we additionally suggest creating a species-specific reference from one of the samples by performing de novo whole genome assembly and extracting the contigs containing rDNA sequence.

In some genomes such as some plants, the morph size may be short enough to be spanned by HiFi reads. In this case ribotin can resolve nearly all variation within the rDNA arrays.

Currently, ribotin has a limitation that copy counts are not estimated. Although the ONT coverages of the morphs are counted, this must be translated to copy counts by the user. Another limitation is that the order of the rDNA morphs is not resolved. This might not be possible with current sequencing technologies due to the size, repetitiveness and homogeneity of the rDNA arrays. Future sequencing technologies combining very long read lengths with high accuracy might make it possible to resolve the order of the rDNA morphs, and therefore assemble rDNA arrays completely. Ribotin also does not assign the morphs to chromosomes, and this must be done manually by the user.

The lack of existing assemblies of complete rDNA morphs makes it difficult to evaluate the accuracy of the results. We have shown that ribotin successfully recovered all major morphs of the CHM13 genome, the so far only human genome assembly which has resolved chromosome specific morphs. Ribotin also had a high accuracy in resolving simulated morphs. However, the other experiments only compared the consistency of ribotin-ref to ribotin-verkko due to a lack of ground truth.

The resolution of rDNA morphs depends on how the rDNA arrays are separated into different tangles. In the CHM13 experiment, manually selecting the five rDNA arrays from the verkko assembly resolved morphs down to an accuracy of five edits, meaning that morphs more similar than five edits are not distinguished. Meanwhile, in ribotin-ref which processes all of the rDNA arrays with the same graph, the accuracy was 80 edits. Although the highly abundant morphs were recovered in both cases, the higher accuracy allowed distinguishing low copy count morphs which are highly similar to the abundant morphs.

Despite the high homogeneity of rDNA arrays, there was noticable variation between the morphs in most of the genomes we tested. In all of the genomes except *C.elegans* there were morphs which are <99% identical to each others. The lengths of the assembled morphs also varied. In CHM13, the shortest morph was over 10 kbp shorter than the longest one. On the other hand, in HG002 the difference was about 5 kbp. The gorilla morphs were relatively more homogenous, with only 1.2 kbp length difference. Even the *A.thaliana* morphs, despite their short average size of around 10 kbp, varied by 3 kbp between the shortest and longest.

Due to chromosome specific variation between rDNA arrays, a single genome wide rDNA consensus might result in a sequence which does not exist in the genome at all. Even though the individual alleles of the consensus sequence at each variant position do exist in the genome, the combination of alleles chosen by the consensus might not. We observed this to be the case in both human genomes we tested as well as the gorilla, where the consensus had at best 99.5% alignment identity with any of the morphs. On the other hand, in *A.thaliana* the consensus exists in the genome although with a relatively low copy count of approximately 25% of the morphs, and in *C.elegans* nearly all morphs are nearly identical to the consensus. This highlights the importance of using resolved, complete rDNA morphs instead of a single genome wide rDNA consensus.

## 5 Conclusion

We have presented ribotin, a tool for assembling rDNA morphs. Ribotin can automatically assemble the most abundant morphs in human genomes, and with a little manual processing, nonhuman genomes as well. Ribotin can enable genomic studies to examine the variation within the rDNA arrays which has so far been difficult to analyze due to their high repetitiveness.

## Supplementary Material

btae124_Supplementary_Data

## Data Availability

No new data were generated or analysed in support of this research.
