# Correction to: Ribotin: automated assembly and phasing of rDNA morphs

**DOI:** 10.1093/bioinformatics/btag126

**Published:** 2026-04-10

This is a correction to: Mikko Rautiainen, Ribotin: automated assembly and phasing of rDNA morphs, *Bioinformatics*, Volume 40, Issue 3, March 2024, btae124, https://doi.org/10.1093/bioinformatics/btae124

The following change has been made to the originally published paper. The **Funding** section statement has been changed to read: “This work was funded by the Academy of Finland Center of Excellence for Complex Disease Genetics (grant number 352793).” instead of: “None declared.”

