# Supplementary material for: Ribotin: automated assembly and phasing of rDNA morphs
Source: Bioinformatics. 2024 Mar 5;40(3):btae124. doi: 10.1093/bioinformatics/btae124 (PMC10948282; doi:10.1093/bioinformatics/btae124)
Supplement: btae124_Supplementary_Data [file btae124_supplementary_data.pdf]

Supplementary Material: Ribotin: Automated assembly and phasing of rDNA morphs

A. Methods

A.1. HiFi Read recruitment

The first step in ribotin-ref is recruiting HiFi rDNA reads. This is done by matching the k-mers of the reference sequence to the reads. The amount of sequence in the read covered by the matching 201-mers is counted, and if the k-mers cover at least 2000 bases, the read is included in the set of rDNA reads.

In ribotin-verkko, the reference sequence is instead matched to nodes in the verkko assembly. 201-mers are matched, and any node with at least 2000 bases covered is a potential rDNA tangle node. Nodes longer than 100kbp are not included as they are considered already well enough resolved. An *rDNA tangle* is a maximal subgraph containing only potential rDNA tangle nodes, with at least 10 nodes, and which contains at least one cycle. Ribotin detects the rDNA tangles using the reference k-mers and graph topology. The user may optionally manually select the rDNA tangles instead of using the default tangle detection. Once the rDNA tangles are found, ribotin recruits HiFi reads which are uniquely assigned to only one tangle, and then runs the next steps separately for each tangle. This step can lose some coverage, since verkko performs graph cleaning which removes low coverage sequences, and discards HiFi reads which are entirely contained within a resolved repeat. We observed that ribotin-verkko typically recruits about 40%-50% fewer HiFi reads than ribotin-ref.

A.2. Graph building and processing

In both ribotin-ref and ribotin-verkko, once the HiFi reads have been recruited, they are processed. First, MBG (Rautiainen and Marschall., 2020b) is used to build a *locally acyclic* de Bruijn graph. We have adjusted MBG to implement a locally acyclic resolution method. In brief, MBG uses the *multiplex de Bruijn graph* algorithm (Bankevich *et al.*, 2022) to resolve nodes by increasing k locally. In the locally acyclic resolution, this multiplex k increase is only done on nodes which are *locally read repetitive*. A

node is locally read repetitive with distance d if it occurs more than once in a read with a distance of at most d between two adjacent occurrences. We set d to be one fifth of the estimated morph size provided by the user. This ensures that there are no small cycles within the graph, while preserving a globally cyclic structure to represent the tandemly repeating nature of the rDNA arrays. MBG uses microsatellite error masking (Rautiainen *et al.*, 2023) to mask away microsatellite indel sequencing errors, which are the second most prevalent sequencing errors in HiFi reads after homopolymer indels. However, some rDNA morphs vary in microsatellite lengths. If the microsatellite length variants are close to other variants, such as SNPs, they will be separated, but if two morphs vary only by microsatellite lengths then this masking artificially homogenizes microsatellite repeats to their most common allele.

Next, ribotin finds a consensus sequence in the graph. The consensus sequence is found with a coverage aware graph walking algorithm. Due to the locally acyclic nature of the graph, any cycle represents one rDNA morph, and the highest coverage cycle is a consensus which picks the highest coverage allele at each bubble. The highest coverage node is selected as the starting point, and then the *generalized widest path* starting and ending at the highest coverage node is selected as the *consensus path*. The *widest path* problem, also called the *maximum capacity path*, is a classical graph problem where edges are given a width, and the desired output is a path with the highest minimum width. The widest path might not be unique as multiple paths can have the same minimum width. The *generalized widest path* further breaks ties by comparing the second smallest width, then third smallest width and so on. The HiFi coverage of the nodes is used as the width of a node, and the HiFi coverage of edges as the width of an edge. This results in a path which maximizes the minimum coverage of both the entire sequence and all of its substrings, essentially picking the most supported allele at each variant site. Since the graph does not have local cycles and the path is constrained to start and end at the same maximum coverage node, the graph can be transformed into a directed acyclic graph by removing any nodes which are in an antichain to the maximum coverage node and duplicating the maximum coverage node as the source and sink node. The widest path problem in directed acyclic graphs can be solved by dynamic programming by iterating the nodes in topological order and storing the minimum width per subpath in $O(V + E)$ time. The generalized widest path problem can similarly be solved by dynamic programming but requires storing all widths encountered so far, and in the worst case comparing all stored widths, resulting in $O(V^2 + VE)$ runtime. This is still fast enough in practice to be insignificant compared to the runtime of other steps of ribotin.

The user may optionally provide a previous reference for orienting the consensus sequence. Matches of unique 101-mers which occur exactly once in both the reference and the consensus are used first to reverse complement the consensus if necessary, and then to rotate it so the two sequences are co-linear. In contrast to the reference used for recruiting reads, the reference used for orienting should contain exactly one full length morph, although it does not need to be from the same individual or species as long as it is similar enough to find 101-mer matches.

Once the consensus sequence is found, variants are detected from the graph. Read subpaths which start at a consensus node, pass through non-consensus nodes and

edges, and end at a consensus node are potential variants. Any variant which is supported by at least three reads is outputed. Ribotin further creates an *allele graph*, which contains the consensus nodes and each variant as a path that starts and ends at a consensus node. Nodes which occur in multiple variants are duplicated to one node per variant.

A.3. Morph resolution

Next, the ultralong ONT reads are processed. The first step is recruiting ONT reads by finding all k-mers in the allele graph, and matching those to the ONT reads. 21-mers are used to count the number of base pairs covered by 21-mers, and if the number of matching base pairs is at least half of the consensus sequence length, the ONT read is included. Note that this step uses the k-mers from the allele graph, not from the reference used for recruiting the HiFi reads. The reason for this is that the allele graph is assumed to be more representative of the genome, and therefore better for recruiting. This step does not consider the copy counts or the locations of the k-mers in the graph, only their presence or absence, and it also does not consider their existence elsewhere in the genome.

The ONT reads are then aligned to the allele graph with GraphAligner (Rautiainen and Marschall., 2020a). In ribotin-ref there is only one allele graph, but in ribotin-verkko there is one graph per tangle. These graphs are concatenated into a single file such that GraphAligner will align simultaneously to all of the graphs without allowing a single alignment to cross over between separate graphs. If an ONT read has alignments to multiple graphs, it is discarded, and if it has alignments only to a single graph it is kept for further processing.

The consensus sequence is used to find morph breakpoints in the allele graph. The 201-mers of the consensus sequence are matched to all nodes to find approximate positions for the morph breakpoints. Then the first and last 50bp of the consensus are aligned to the approximate positions to find exact morph breakpoints.

The morph breakpoints are used to extract *loops* from the ONT reads. Whenever the alignment of an ONT read crosses two morph breakpoints, it has completed a single traversal around the allele graph and the path is extracted as a loop. Every loop represents one complete sequence of an rDNA morph, potentially with sequencing errors. All such complete loops are collected from the ONT alignments. Partial loops at the start or end of a read are ignored. We ignore the sequence of the read itself and use the sequence of the path in the graph as the *loop sequence* of a loop. The reason for using the sequences of the graph path instead of the read is that aligning the ONT reads to the graph implicitly performs error correction and enables the reads to be clustered with a more stringent identity threshold.

The loops are then clustered based on sequence similarity. The loops are aligned in an all-vs-all manner using global alignment with the wavefront alignment algorithm (Marco-Sola *et al.*, 2020). Any pair of loops with an edit distance less than the maximum cluster difference d (adjustable by user, by default 200 edits corresponding to 99.6% identity) are merged into the same cluster using a union-find data structure. We call these clusters the *rough clusters*. The rough clusters usually contain multiple

morphs per cluster, and have the property that any pair of loops between different rough clusters has an edit distance at least d. The converse is not true and pairs of loops within the same rough cluster can have any edit distance, including more than d.

The rough clusters are then further refined. We cluster the loops by considering each loop a single point, with the distance between points defined by the edit distance of their loop sequences. The ground truth of this clustering problem is that each morph corresponds to one cluster such that there is an unknown number of clusters, the clusters have a different number of points due to their different copy counts, the clusters have a small dense center composed of low error rate points and a large sparse ring of high error rate outliers, and different clusters can be close enough to be indistinguishable if their variation is small compared to the average read error rate.

We use the density based dbscan algorihm (Ester *et al.*, 1996) for clustering since it does not require the number of clusters to be known, and its parameters can be estimated from the data. The dbscan algorithm requires two parameters: a maximum distance ϵ, and a minimum number of points $minPts$, and then it classifies the points into *core points*, *border points* and *outliers* depending on the number of points close to them. If a point has at least $minPts$ points within distance ϵ, it is a core point. Any pair of core points within ϵ distance of each other belong in the same cluster. If a point is not core but is within ϵ of a core point, it is a border point. Otherwise a point is an outlier point. In the basic dbscan algorithm, border points may belong to different clusters depending on the order of iteration and so their assignment can be ambiguous. We detect this case and assign border points to a cluster only if their assignment is unambiguous, that is, all core points within distance ϵ are part of the same cluster. Ambiguous border points and outlier points are discarded. We call these clusters the *density clusters*.

The intuition for estimating ϵ is that the edit distance histogram between all pairs of loops is multimodal: there is one mode for all loop pairs within the same morph, and one mode for each pair of different morphs. Importantly, since the edit distance between intra-morph loop pairs is composed only of read sequencing errors, the average edit distances of intra-morph loop pairs are the same in all clusters and they are all part of the same mode. We estimate the average intra-morph edit distance by aligning all pairs of loop sequences in the same rough cluster to each others. The peak in the loop pair edit distance histogram is then taken as the average intra-morph edit distance and the ϵ parameter. Ribotin further has a parameter for minimum ϵ (by default 5) which limits how low the ϵ parameter may be chosen. If the edit distance peak is below minimum ϵ, then ϵ is chosen as the parameter instead of the edit distance peak. The $minPts$ parameter should be chosen based on the expected number of loops per a single copy morph such that there is a high probability that a single copy morph has at least $minPts$ loops. This could be estimated from the genomic coverage and read length distribution, but we use a constant 5 as $minPts$ since high copy count morphs will almost certainly have more than 5 loops.

Due to the density based method of dbscan, the same cluster might contain points which are further apart than ϵ. This can happen if several highly similar morphs are more similar than ϵ, since the cluster will be the transitive closure of the morphs.

Therefore the parameter ϵ should not be interpreted as a strict limit on the morph resolution, but instead as a guideline on what kind of divergence is resolved, and a single cluster can contain morphs which differ by more than ϵ.

Once the loops have been clustered with dbscan, the clusters are then further phased with a heuristic method. The heuristic is based on a simple idea: any sequencing errors will be independent between loops, while valid variation between morphs will co-occur between different loops. If there are two bubbles which split the loops into the same two bipartitions, and both sets in the bipartition are covered by at least 10 loops, then the two sets are separated into their own clusters. This is recursively repeated until the heuristic no longer splits the cluster. This method is very stringent due to requiring an exact bipartition between the two bubbles, and even a single loop misaligning on one of the bubbles will prevent the phasing heuristic from working. It also does not take into account more complicated situations involving multiple variants.

Finally, the consensus for each cluster is generated. For each cluster, the subgraph induced by the loop paths is considered, and the coverages of its nodes and edges are taken from the loop paths. A consensus path is generated based on the loop paths in the cluster by taking the most abundant allele at each bubble, and the sequence of the consensus path is outputed as the morph sequence, and the number of loops contained in the cluster as the coverage. These sequences are the final morph consensuses outputed by ribotin.

Ribotin also outputs a *morph graph* which represents the possible adjacencies between the morphs. The nodes of the morph graph are the morphs, and edges are added whenever at least two ONT reads have the morphs adjacent to each others. The morph graph also contains the paths of the ONT reads through the morph graph. This morph graph did not enable resolving the complete order of the morphs in any of our experiments, but it can be used to get some subsets of the order when there are particularly long ONT reads.

B. Results

B.1. Human rDNA simulation

Generating biologically plausible rDNA morphs requires knowledge on what kind of variants can occur in the rDNA, including structural variation. We generated simulated morphs using the nine so-far assembled complete rDNA morphs: the eight major morphs of the CHM13 assembly (Nurk *et al.*, 2022) and the KY962518.1 rDNA reference sequence (Kim *et al.*, 2018).

First, we generated *mosaic sequences* of the nine existing rDNA morphs. We detected *core sequences* in the morphs, sequences which are present in all nine morphs and only occur once per morph. To do this we built a morph pangenome graph by aligning the morphs against each other with minimap2, then used seqwish to collapse together the homologous areas. Any node which occurs exactly once in all morph paths in the graph represents core sequence. Then, we sampled mosaic sequences: first, start at a random morph and add its sequence until a core sequence is reached.

Then, at each core sequence, switch to a random morph and add its sequence until the next core sequence. Repeating this until the end of a sequence is reached generates a sequence which is a random mosaic of the input morphs. Empirically, this results in an average divergence of 4.2% between morphs.

After the mosaic sequences were generated, we added random mutations to them. We inserted random substitutions at a rate of 0.1%, random insertions of length 1-10bp at rate 0.01%, and random deletions of length 1-10bp at rate 0.01%, which adds an average divergence of 0.2% due to random mutations.

We randomly assigned a copy count uniformly from 1 to 30 copies to each simulated morph. The morphs were concatenated into a single simulated rDNA array by duplicating each morph according to its copy count. Finally, we added 100kbp of random sequence to the start and end of the simulated rDNA array to prevent any edge effects from biasing the read coverages of the morphs.

Each rDNA array was simulated with 20 morphs, resulting in an average of 300 copies of rDNA, or approximately 13.5Mbp of sequence, per simulated rDNA array.

Once the simulated rDNA arrays were generated, we used pbsim3 (Ono *et al.*, 2022) to simulate HiFi and ultralong ONT reads from them. For ONT reads, we used the command "pbsim –strategy wgs –method qshmm –qshmm QSHMM-ONT-HQ.model –depth 30 –genome rDNA_sequence_ground_truth.fa –length-mean 40000 –length-sd 30000", and for HiFi reads, we first used the command "pbsim –strategy wgs –method qshmm –qshmm QSHMM-RSII.model –depth 10 –genome rDNA_sequence_ground_truth.fa –pass-num 10 –length-mean 15000 –length-sd 2000" to generate the subreads and then the command "ccs sd_0001.bam simulated_hifi.bam" to generate HiFi reads from the subreads. This corresponds to the default accuracy parameters, read lengths which represent current ultralong ONT and HiFi read length distributions, and sequencing depth approximately matching one flow cell of HiFi on PacBio Sequel II and one flow cell of ultralong ONT on Promethion.

We ran ribotin-ref on the simulated reads. To compare the resulting morphs with the ground truth simulated morphs, we first aligned them with minimap2, and filtered the alignments to those with at least 99% identity covering at least 99% of both morphs. For evaluating the sensitivity and specificity, we divide the morphs into three cases: a *true positive* is a simulated morph which aligns to a ribotin-ref morph, a *false negative* is a simulated morph which does not align to a ribotin-ref morph, and a *false positive* is a ribotin-ref morph which does not align to a simulated morph.

For evaluating the correspondance between ribotin-ref coverages and copy counts, the morphs must be uniquely matched. We picked only those pairs of simulated and ribotin-ref morphs which align to each others and to no other morphs. Similarly, for evaluating the error rate, we measured only the same uniquely matched pairs and measured the average divergence of the alignments, assuming that any unaligned sequence is mismatched.

B.2. C. elegans

We ran ribotin-ref with estimated size of a single morph 10000, rough clustering maximum edit distance $d = 10$, and density clustering minimum $\epsilon = 1$ and inputing the

HiFi reads as both HiFi reads and ONT reads. We ran the two strains separately. The ALT1 strain resulted in two morphs, one of which had a 3478bp deletion but otherwise had identical sequence. We found several HiFi reads which supported the deletion. The shorter morph's coverage (13 reads) is consistent with a copy count of 1 or 2 but an another method would be required to find the exact copy count. The ALT2 strain resulted in a single morph with identical sequence to the ALT1 strain's major morph but rotated by 625bp and reverse complemented, since we did not input a previous reference for orienting the morphs.

Ribotin reported 20 variants in the ALT2 strain. Supplementary Table 2 reports the positions and the sequences of the variants. 17 of the variants are individual SNPs and three (IDs 3, 11 12) are variants where a pair of SNPs was merged into the same variant, totaling 23 SNPs. Two of the variants which merged a pair of SNPs coincided with the highest coverage variant (ID 10 at position 5051). Although HiFi reads have a systematically higher error rate for homopolymer and microsatellite indels, the highest coverage SNP could not have been created by indel errors. The three variants which merge a pair of SNPs also look unlikely to be sequencing errors, since sequencing errors would be independently distributed and therefore would lead to reads which have just one of the SNPs. For example, the variant id 3 has two SNPs, but if they were sequencing errors we would expect to see both of the individual SNPs at a higher coverage than the combination of the two SNPs. Variants 11 and 12 also merge a pair of SNPs and they do coincide with a SNP at their start, but here again we would expect to see some reads support the SNP at their end without the SNP at the start if the variants were sequencing errors. The lack of those single SNPs is evidence that the variants which report a pair of SNPs are indeed real variation and not just sequencing errors. The variants which contain a single SNP cannot be confidently said to be real or false positives, since the extremely high coverage of the rDNA arrays means that even very low probability systematic errors will have read support. For example, a hypothetical systematic error which occurs at a rate of 0.5% would be expected to be supported by 11 reads in ALT2.

The total coverage of the morphs in ALT1 is 947, and in ALT2 is 2118. This implies that the copy counts of ALT2 should be slightly more than twice that of ALT1. We checked whether this might be an artifact of ribotin's methods by using two other methods to estimate the rDNA coverage of the same set of HiFi reads. First we used MBG to build graphs of the two strains separately, and found the locations of the rDNA tangles in those graphs. Then we found the highest coverage nodes in the tangles, which were 94x and 251x for ALT1 and ALT2 respectively, implying that ALT2 has about 2.5x more rDNA HiFi reads than ALT1. Next we used minimap2 to align all the ALT1 and ALT2 reads to ribotin's consensus rDNA sequence with the parameters "minimap2 -eqx -x asm5 -c ribotin-reference.fa hifi-reads.fa". We filtered the alignments to those with length at least 3000bp to remove false positives from reads originating from elsewhere in the genome and measured the peak coverage at any point in the reference. This resulted in peak coverage 1910 and 4046 for ALT1 and ALT2 respectively, consistent with ALT2 rDNA hifi reads being about twice as abundant as ALT1 rDNA hifi reads. The ratios between ALT1 and ALT2 are similar in all three methods, but the absolute numbers differ by a large amount. This is explained by

the different methods: MBG requires long exact k-mer matches where any sequencing errors prevent a read from being counted, ribotin requires an alignment which spans an entire morph from the specific start position to the end position and therefore loses a noticable amount of shorter reads while allowing sequencing errors, and minimap2 requires a 3000bp alignment which loses almost no reads due to length while allowing sequencing serrors.

The whole genome hifi dataset of ALT1 (resp. ALT2) has haploid coverage 15x (13x), with expected 8x (7x) coverage of complete single morphs. Naively applying the expected coverage of complete morphs to the ribotin-ref morphs implies an rDNA copy count of 118 for ALT1 and 302 for ALT2. Applying the average haploid coverage to the minimap2 coverages implies an rDNA copy count of 127 for ALT1 and 311 for ALT2. These estimates are roughly consistent with each others and previous C. elegans rDNA copy count estimates, but a more accurate method would be required to get the exact copy count. In addition, since the rDNA copy count varies greatly between the two strains, it is plausible that the copy counts might vary between individuals of the same strain as well, implying that instead of a single copy count per strain, a distribution of copy counts would be a more meaningful concept.

Assuming a copy count of 300 for ALT2 would imply the rDNA array is about 2.16Mbp long. Further assuming that all 23 SNPs are real would imply a SNP on average every 94kbp. This is within the lengths routinely reachable by ultralong ONT reads. However, any uneven distribution of the SNPs could make the distances much longer, and it is unlikely that all 23 SNPs are real variation instead of systematic sequencing errors. In addition, since the small amount of DNA in each C. elegans individual means that sequencing must be done on a pool of multiple worms instead of a single individual, resolving the rDNA array by connecting SNPs would require that all of the sequenced individuals have the same rDNA sequence with the same SNPs in the same order. Assuming a copy count of 120 for the ALT1 strain would imply an rDNA array length of about 860kbp. Current ultralong ONT sequencing already generates a small number of outlier reads of several hundreds of base pairs, and the current record for the longest individual nanopore read is a few megabases long. It is plausible that near future sequencing reads could be long enough to routinely span through entire C. elegans rDNA arrays.

C. Supplementary figures

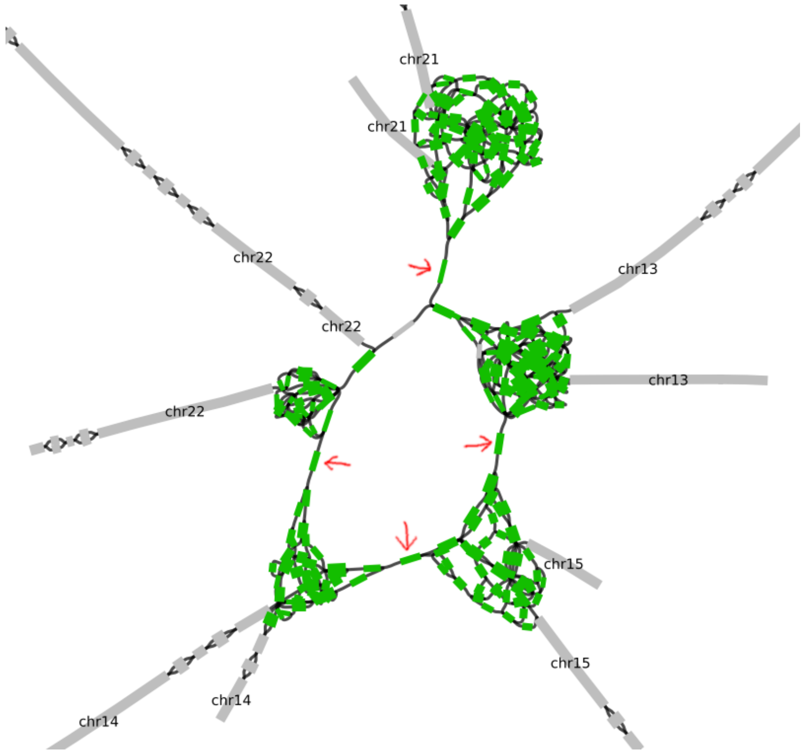

Figure 1: Bandage (Wick *et al.*, 2015) plot of the verkko version 1.4.1 assembly of CHM13, zoomed in on the rDNA arrays. The green nodes were classified as rDNA tangle nodes by ribotin. The gray nodes are labeled according to their unique alignment to the CHM13 reference. The five rDNA arrays are clearly distinct, but spurious nodes connecting the tangles (red arrows) caused all five arrays to be assigned to the same tangle.

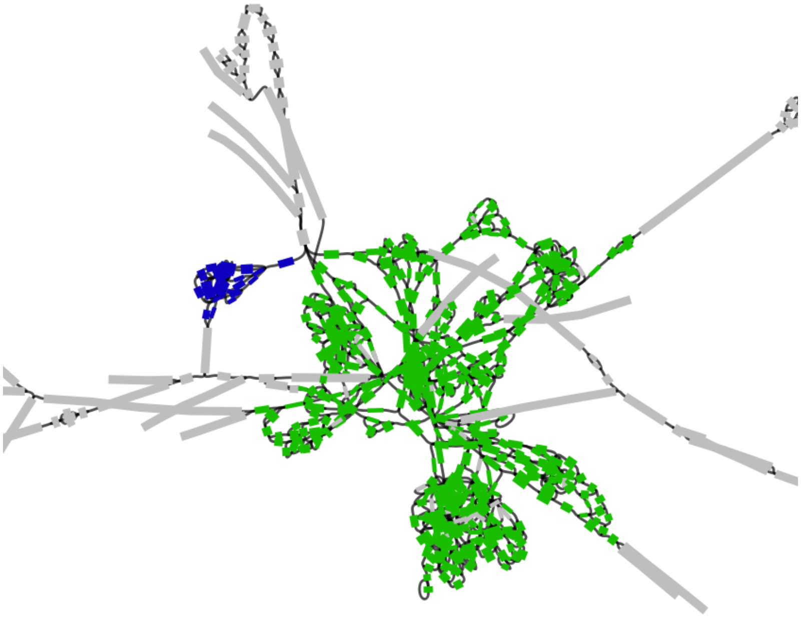

Figure 2: Bandage (Wick *et al.*, 2015) plot of the verkko assembly of HG002, zoomed in on the rDNA arrays. The green nodes were classified as one rDNA tangle by ribotin, and the blue nodes were classified as a second rDNA tangle. Visually there appear to be several arrays which were all assigned to the same green tangle.

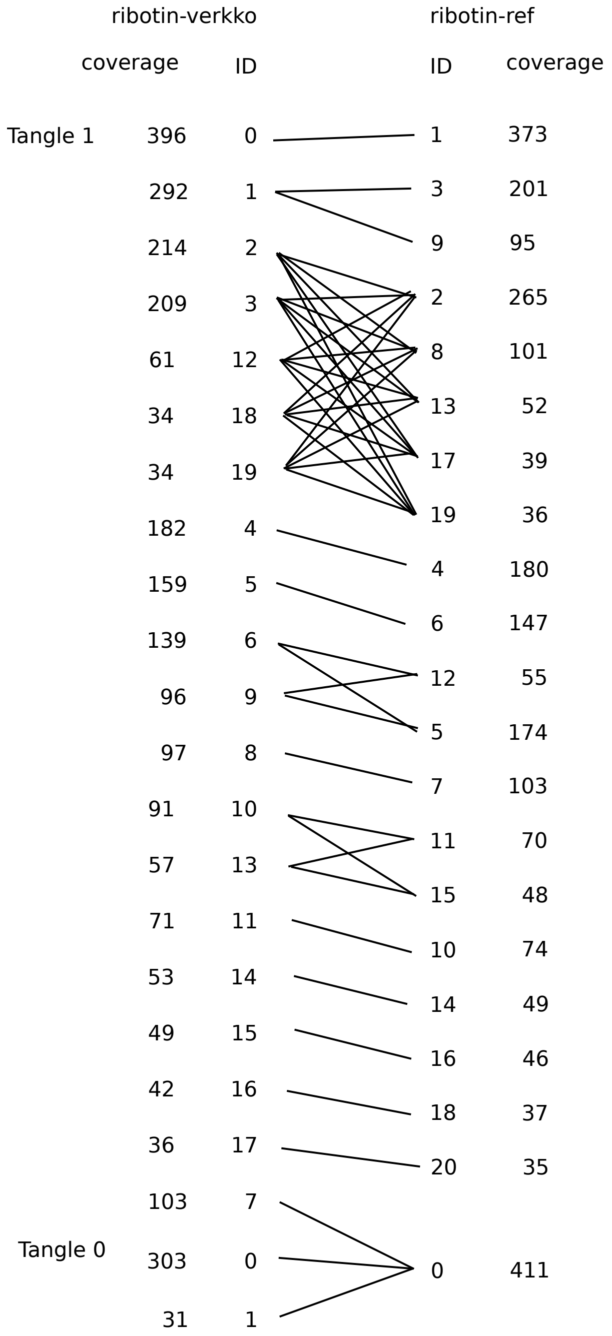

Figure 3: Comparison of ribotin-verkko with automatic rDNA tangle detection and ribotin-ref morphs for HG002. Morphs with coverage less than 30 are not shown. All matching morphs are connected by lines. The ribotin-verkko morphs are grouped by tangle, and both ribotin-ref and ribotin-verkko morphs are ordered for clarity.

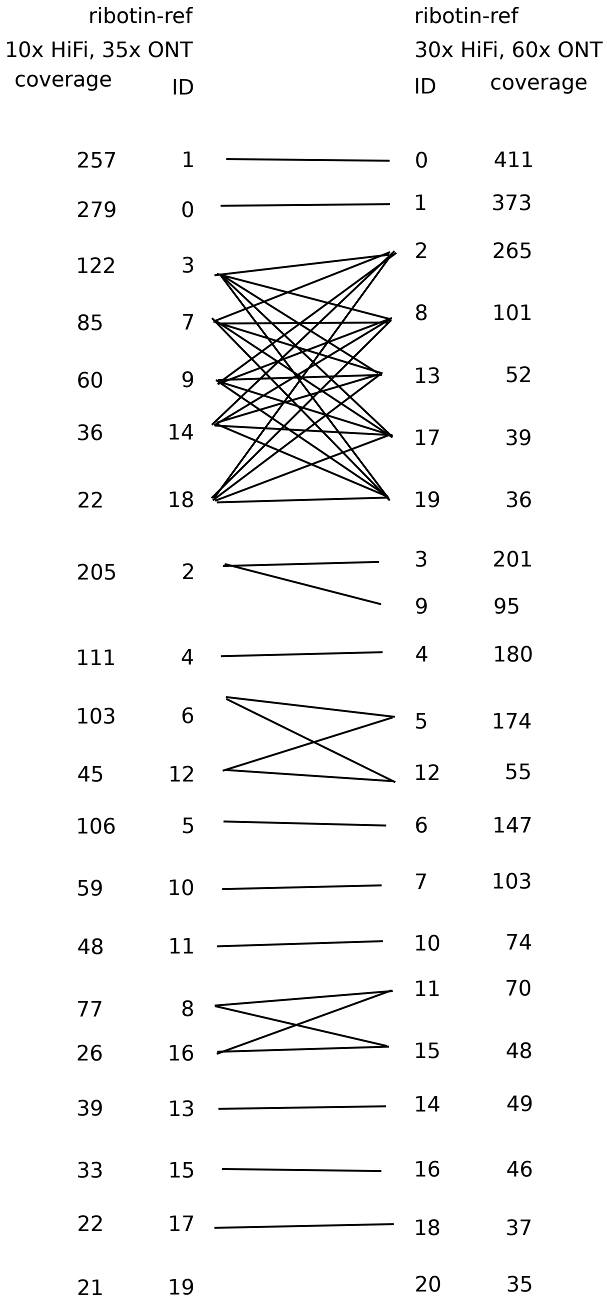

Figure 4: Comparison of ribotin-ref with high and low coverage for HG002. Morphs with coverage less than 30 for high coverage and 20 for low coverage are not shown. All matching morphs are connected by lines. The morphs are ordered for clarity.

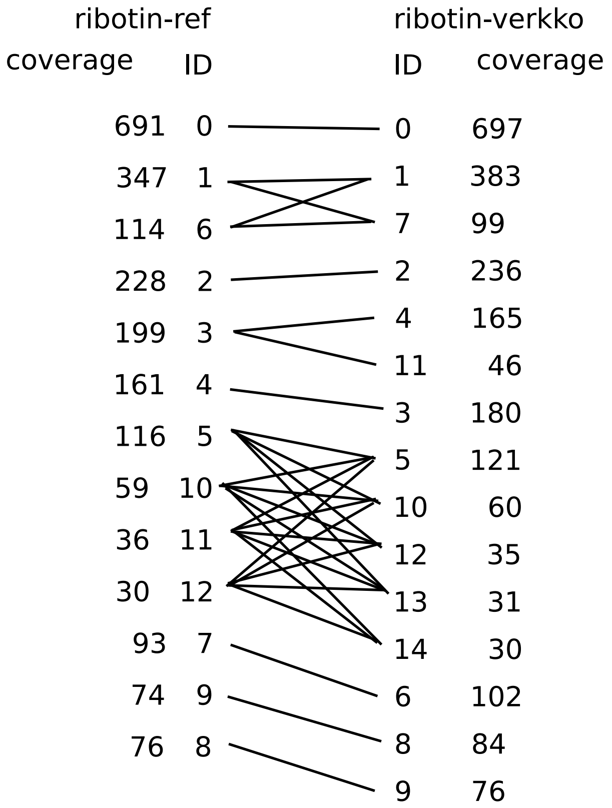

Figure 5: Comparison of ribotin-verkko with automatic rDNA tangle detection and ribotin-ref morphs for CHM13. Morphs with coverage less than 30 are not shown. All matching morphs are connected by lines. The morphs are grouped and ordered for clarity.

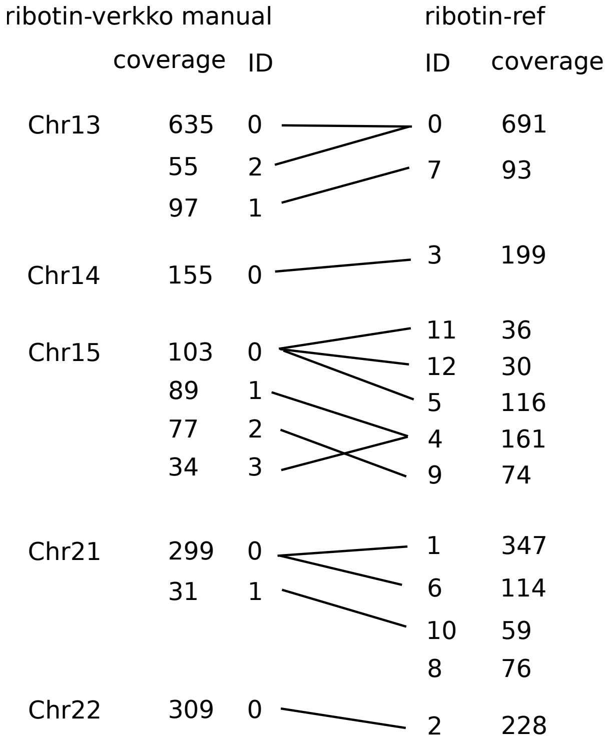

Figure 6: Comparison of ribotin-verkko with manually selected rDNA tangles and ribotin-ref morphs for CHM13. Morphs with coverage less than 30 are not shown. All matching morphs are connected by lines. The ribotin-verkko morphs are grouped by tangle, and both ribotin-ref and ribotin-verkko morphs are ordered for clarity. Ribotin-ref does not group the morphs in any way and the whitespaces are only for clarity. Ribotin-ref morph ID 8 matched several ribotin-verkko morphs in chromosome 21 with coverage less than 30, and many of the ribotin-ref morphs matched to ribotin-verkko morphs with coverage less than 30.

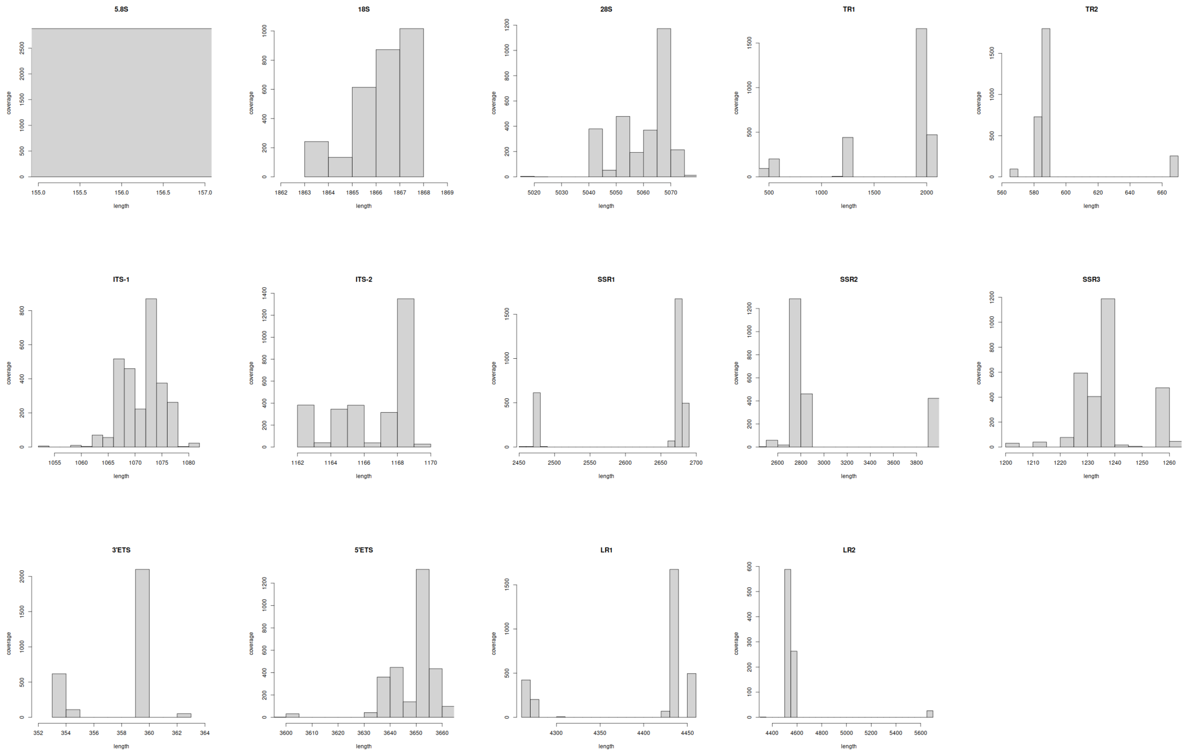

Figure 7: Length histogram of different locuses in the HG002 ribotin-ref morphs. The lengths in the x-axis are extracted from the morph annotations and the y-axis counts are from the count of ONT reads which support each morph.

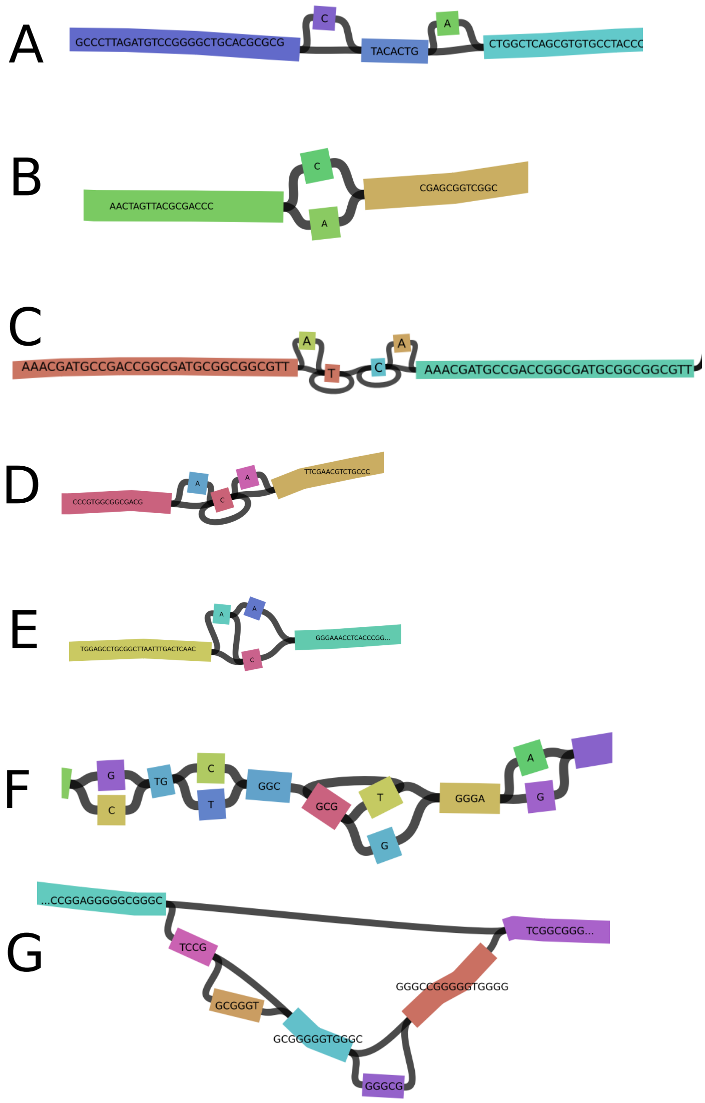

Figure 8: Bandage plots of the HG002 morph pangenome zoomed in to examples of variant sites, with the nodes labeled by their sequences. A: Two small indels in the 18s gene. B: A SNP in the 18s gene. C, D, E: Multiallelic sites in the 18s gene, showing varying homopolymer lengths combined with small indels. F: A multilallelic site in the 5' ETS showing variation in a short tandem repeat length with the repeat motif GCGT with three SNPs nearby. G: A multiallelic site in the 28s gene with nested indels.

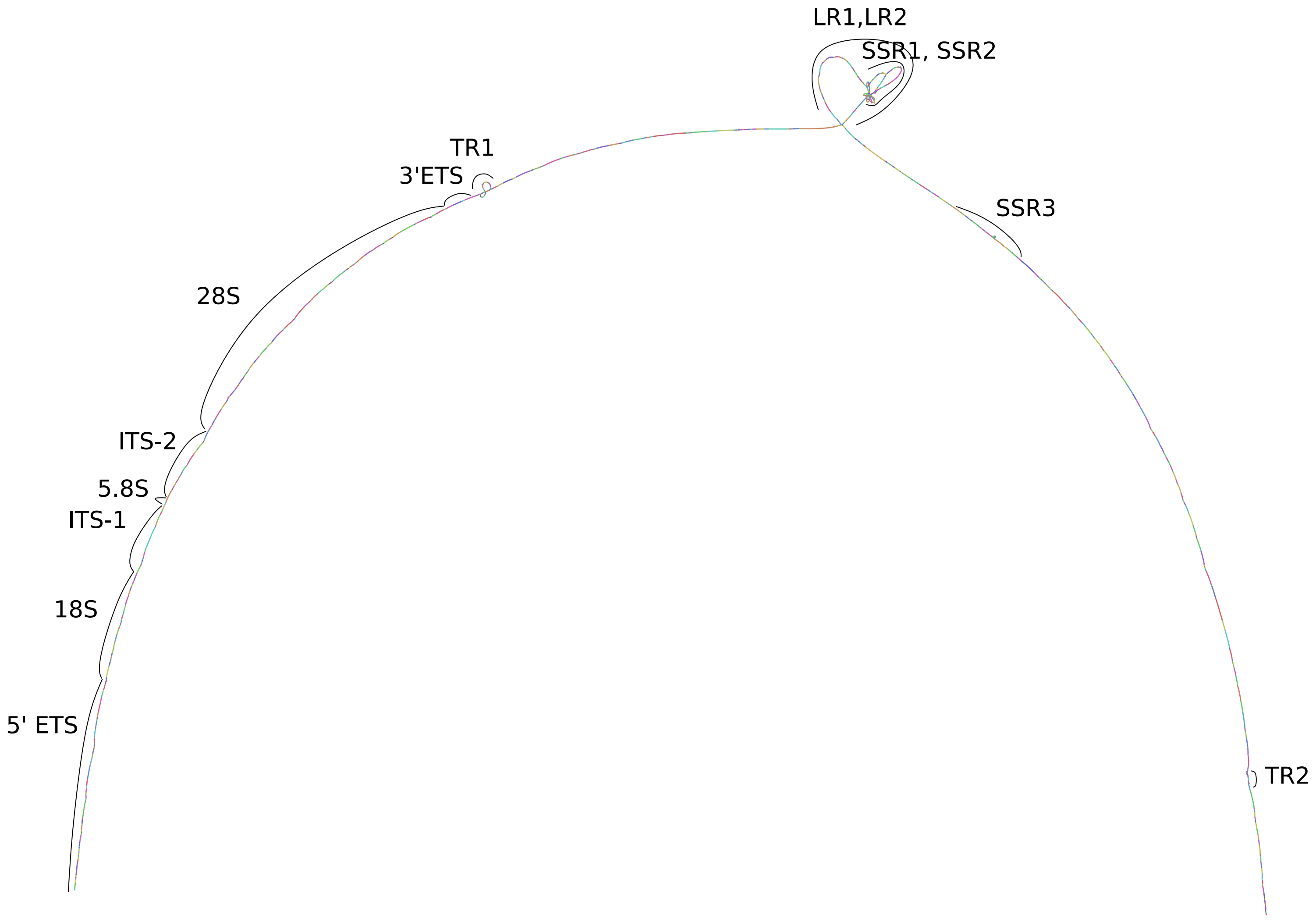

Figure 9: Bandage plot of the morph pangenome of HG002 with some regions labeled.

D. Supplementary tables

Locus	Biallelic SNPs	Biallelic indels	Multiallelic sites	Total
5'ETS	46	32	7	85
18s	7	37	7	51
ITS-1	10	7	5	22
5.8s	2	0	0	2
ITS-2	11	11	6	28
28s	29	59	17	105
3'ETS	1	1	2	4

Table 1: Number of variants in and near the genes of the HG002 morph pangenome.

ID	Location in consensus	Ref	Alt	Alt coverage
0	1171	G	A	12
1	1372	T	C	3
2	1580	C	T	5
3	1910-1914	GTGG	ATGA	5
4	3213	C	T	10
5	3453	C	T	4
6	3910	T	A	4
7	4097	T	A	10
8	4136	A	C	23
9	4163	C	T	19
10	5051	G	T	66
11	5051-6630	GAAAGG...TTGCAA	TAAAGG...TTGCAG	4
12	5051-6679	GAAAGG...TCCTCG	TAAAGG...TCCTCA	12
13	5192	A	T	5
14	5565	A	T	5
15	6128	G	A	10
16	6259	A	T	21
17	6534	G	C	4
18	6745	T	A	12
19	7189	G	C	4

Table 2: Variants reported by ribotin-ref in the C.elegans ALT2 strain with ribotin's consensus sequence as the reference. The ref and alt sequences in variant ID 11 are identical except for the first and last base, and similarly in variant ID 12.

References

Bankevich, A., Bzikadze, A. V., Kolmogorov, M., Antipov, D., and Pevzner., P. A. (2022). Multiplex de bruijn graphs enable genome assembly from long, high-fidelity reads. *Nature Biotechnology*.

Ester, M., Kriegel, H.-P., Sander, J., and Xu., X. (1996). A density-based algorithm for discovering clusters in large spatial databases with noise. *Proceedings of the Second International Conference on Knowledge Discovery and Data Mining*.

Kim, J.-H., Dilthey, A. T., Nagaraja, R., Lee, H.-S., Koren, S., Dudekula, D., Iii, W. H. W., Piao, Y., Ogurtsov, A. Y., Utani, K., Noskov, V. N., Shabalina, S. A., Schlessinger, D., Phillippy, A. M., and Larionov, V. (2018). Variation in human chromosome 21 ribosomal rna genes characterized by tar cloning and long-read sequencing. *Nucleic Acid Research*.

Marco-Sola, S., Moure, J. C., Moreto, M., and Espinosa., A. (2020). Fast gap-affine pairwise alignment using the wavefront algorithm. *Bioinformatics*.

Nurk, S., Koren, S., Rhie, A., Rautiainen, M., Bzikadze, A. V., Mikheenko, A., Vollger, M. R., Altemose, N., Uralsky, L., Gershman, A., Aganezov, S., Hoyt, S. J., Diekhans, M., Logsdon, G. A., Alonge, M., Antonarakis, S. E., Borchers, M., Bouffard, G. G., Brooks, S. Y., Caldas, G. V., Chen, N.-C., Cheng, H., Chin, C.-S., Chow, W., de Lima, L. G., Dishuck, P. C., Durbin, R., Dvorkina, T., Fiddes, I. T., Formenti, G., Fulton, R. S., Fungtammasan, A., Garrison, E., Grady, P. G., Graves-Lindsay, T. A., Hall, I. M., Hansen, N. F., Hartley, G. A., Haukness, M., Howe, K., Hunkapiller, M. W., Jain, C., Jain, M., Jarvis, E. D., Kerpedjiev, P., Kirsche, M., Kolmogorov, M., Korlach, J., Kremitzki, M., Li, H., Maduro, V. V., Marschall, T., McCartney, A. M., McDaniel, J., Miller, D. E., Mullikin, J. C., Myers, E. W., Olson, N. D., Paten, B., Peluso, P., Pevzner, P. A., Porubsky, D., Potapova, T., Rogaev, E. I., Rosenfeld, J. A., Salzberg, S. L., Schneider, V. A., Sedlazeck, F. J., Shafin, K., Shew, C. J., Shumate, A., Sims, Y., Smit, A. F. A., Soto, D. C., Sovic, I., Storer, J. M., Streets, A., Sullivan, B. A., Thibaud-Nissen, F., Torrance, J., Wagner, J., Walenz, B. P., Wenger, A., Wood, J. M. D., Xiao, C., Yan, S. M., Young, A. C., Zarate, S., Surti, U., McCoy, R. C., Dennis, M. Y., Alexandrov, I. A., Gerton, J. L., O'Neill, R. J., Timp, W., Zook, J. M., Schatz, M. C., Eichler, E. E., Miga, K. H., and Phillippy, A. M. (2022). The complete sequence of a human genome. *Science*.

Ono, Y., Hamada, M., and Asai., K. (2022). Pbsim3: a simulator for all types of pacbio and ont long reads. *NAR Genomics and Bioinformatics*.

Rautiainen, M. and Marschall., T. (2020a). GraphAligner: rapid and versatile sequence-to-graph alignment. *Genome Biology*.

Rautiainen, M. and Marschall., T. (2020b). MBG: Minimizer-based sparse de Bruijn Graph construction. *Bioinformatics*.

Rautiainen, M., Nurk, S., Walenz, B. P., Logsdon, G. A., Porubsky, D., Rhie, A., Eichler, E. E., Phillippy, A. M., and Koren., S. (2023). Telomere-to-telomere assembly of diploid chromosomes with verkko. *Nature Biotechnology*.

Wick, R. R., Schultz, M. B., Zobel, J., and Holt, K. E. (2015). Bandage: interactive visualization of de novo genome assemblies. *Bioinformatics*.
